# Urinary bladder collision tumors: A case report and literature review

**DOI:** 10.3892/mi.2025.225

**Published:** 2025-03-07

**Authors:** Sami S. Omar, Saman S. Fakhralddin, Rawa M. Ali, Ari M. Abdullah, Soran H. Tahir, Bryar Othman Muhammed, Fahmi H. Kakamad, Abdullah A. Qadir, Hiwa O. Abdullah, Berun A. Abdalla, Suhaib H. Kakamad, Jihad Ibrahim Hama

**Affiliations:** 1 Kscien Organization for Scientific Research (Middle East Office), Sulaymaniyah 46001, Iraq; 2 Rizgary Oncology Center, Erbil 44001, Iraq; 3 Department of Scientific Affairs, Smart Health Tower, Sulaymaniyah 46001, Iraq; 4 College of Medicine, University of Sulaimani, Sulaymaniyah 46001, Iraq; 5 Department of Pathology, Sulaimani Surgical Teaching Hospital, Sulaymaniyah 46001, Iraq; 6 Smart Health Tower Raparin, Rania, Sulaymaniyah 46001, Iraq; 7 Research Center, University of Halabja, Halabja 46018, Iraq

**Keywords:** collision tumor, urinary bladder, adenocarcinoma, squamous cell carcinoma

## Abstract

A collision tumor of the urinary bladder refers to a very rare condition in which two distinct types of tumors occur simultaneously within the same bladder. The present study reports the case of a patient with a collision tumor in the urinary bladder. A 43-year-old female patient was referred following a diagnosis of bladder carcinoma. The patient had previously undergone a cystoscopy-directed transurethral resection of a bladder tumor (TURBT) to remove a mass from the urinary bladder. A biopsy revealed that the tumor was a moderately differentiated T2-adenocarcinoma. A contrast-enhanced computed tomography (CT) scan revealed an irregular, thickened bladder wall. Based on the PET scan result and the initial TURBT result, which revealed T2-stage adenocarcinoma, a radical cystectomy was recommended. The final histopathological diagnosis was poorly differentiated adenocarcinoma (pT2 N0 M0) and well-differentiated squamous cell carcinoma T1 N0 M0 as a collision tumor. In addition, the present study also provides a literature review which summarizes cases of urinary bladder collision tumors in male patients aged 50 to 74 years, primarily presenting with hematuria. Diagnoses were confirmed using imaging techniques, such as CT scans and MRIs. Surgical treatments, including TURBT and radical cystectomy, are commonly used, with some cases also receiving chemotherapy or immunotherapy. Outcomes varied, with some patients experiencing recurrence or metastasis, while others remained recurrence-free post-surgery. A bladder collision tumor is a rare disease; extensive radiological study and careful examination of the biopsy are required to diagnose the condition.

## Introduction

Collision tumors refer to the occurrence of two distinct tumors within the same organ or mass, each with separate cellular lineages and genetic origins, but lacking a visible transitional zone between them. This phenomenon is most commonly observed in organs such as the liver, stomach, adrenal glands, ovaries, lungs, kidneys and colon (1).

A collision tumor of the urinary bladder refers to a very rare condition in which two distinct types of tumors occur simultaneously within the same bladder. These tumors may have different origins and growth patterns, and they may be benign or malignant. The coexistence of two different tumor types in the same location is what distinguishes a collision tumor from a composite tumor, in which different cell types from the same tissues are involved (2,3). The most common combination observed in bladder cancer collisions is the concurrent presence of urothelial carcinoma, which is the most common subtype of malignant bladder tumors, and another type of tumor, such as adenocarcinoma, squamous cell carcinoma (SCC) and other subtypes (4,5). Urothelial carcinoma is the most common primary histological type involving the urinary bladder, which comprises 90 to 95% of the primary malignancies, while the other non-urothelial carcinomas, e.g., adenocarcinoma, SCC, neuroendocrine carcinoma and lymphoma, are uncommon and often diagnostically challenging (6,7). Both adenocarcinoma and SCC account for 0.5-2% and 1-3% of the primary bladder malignancies, respectively (8,9). Oncogenic factors, such as p53, VEGF and EGFR, are known to play critical roles in the signal transduction pathways of collision tumors. Based on whether cancer cells can infiltrate the muscular layer of the bladder, bladder cancer is divided into two subtypes, namely non-muscle invasive bladder cancer (NMIBC) and muscular invasive bladder cancer (MIBC). The transurethral resection of bladder tumor (TURBT) is the primary treatment modality for NMIBC (10).

The present study reports and discusses a very rare case of a bladder collision tumor. The references have been inspected for reliability, and the report has been written according to the CaReL guidelines (11,12).

## Case report

### Patient information

A 43-year-old female patient with a history of vesical stones was referred to the Rzgary Oncology Clinic at Rzgary Teaching Hospital (Erbil, Iraq) after being diagnosed with bladder carcinoma.

### Clinical findings

**Figure 1:**
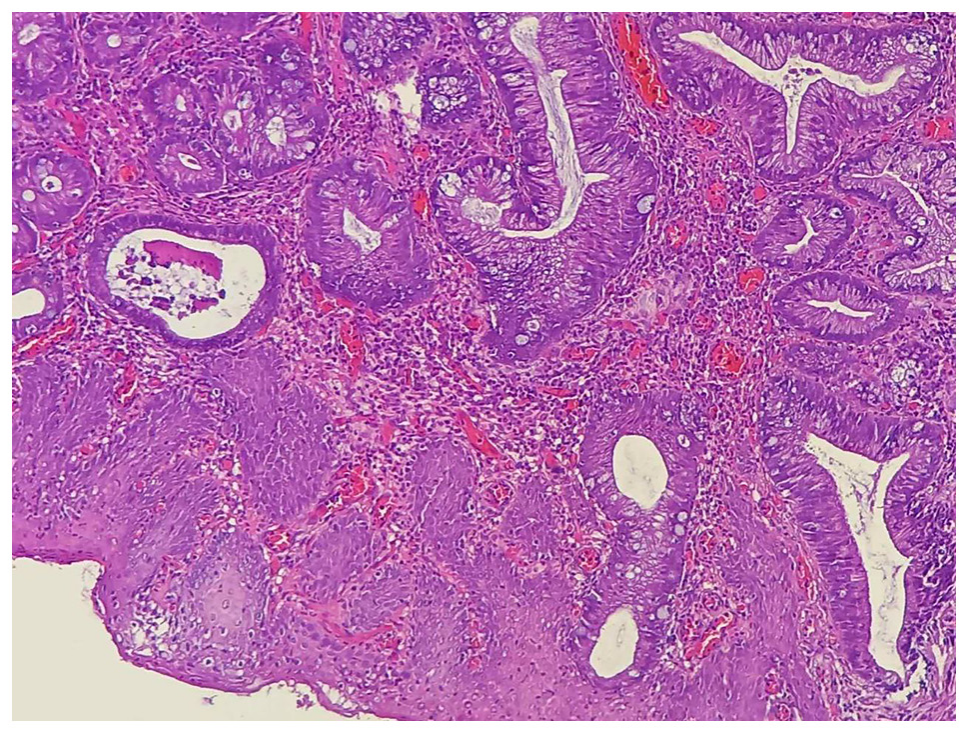
The stained section indicates numerous fragments of bladder tissue heavily involved by moderately differentiated adenocarcinoma.

The medical history of the patient included controlled diabetes mellitus, hypertension and multiple sessions of cystoscopic laser lithotripsy, as well as open vesical stone extractions. She had previously undergone a cystoscopy-directed TURBT to remove a mass from the urinary bladder, and a histopathological examination of the biopsy (performed at Rzgary Oncology Laboratory), identified the tumor as a moderately differentiated T2 adenocarcinoma (Fig. 1). Following this, the patient was referred to the Smart Health Tower (Sulaymaniyah, Iraq) for further management.

### Diagnostic assessment

**Figure 2:**
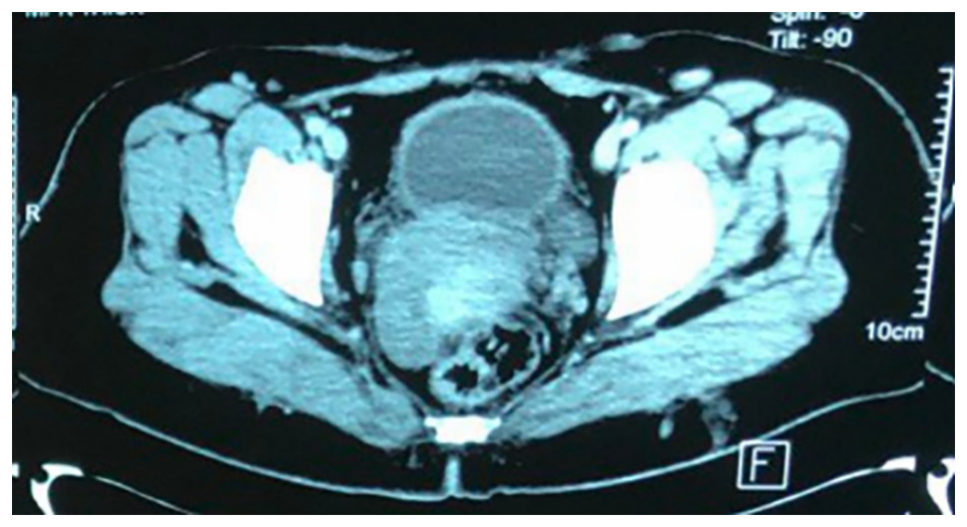
A contrast-enhanced CT scan of the pelvis indicating a mildly thickened urinary bladder wall; however, no obvious mass is present, and the bladder appears small in size.

**Figure 3:**
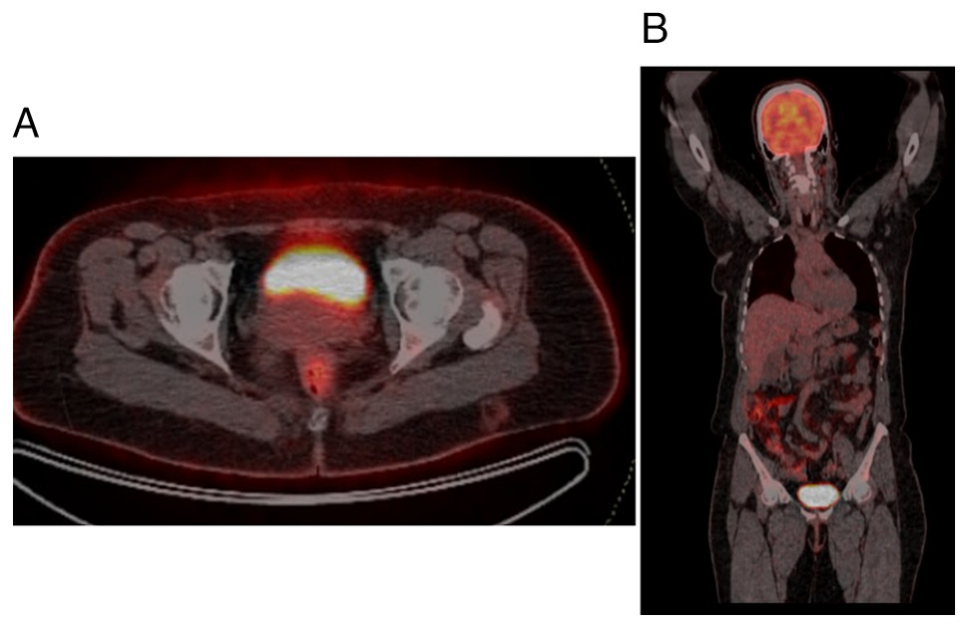
(A) PET-CT axial sections of the pelvis illustrating that radioactive substances are excreted in the urinary bladder with no abnormal uptake in the pelvis. (B) Whole-body PET-CT coronal section shows normal uptake in the brain and intestine, excretion in the urinary bladder, and no metastasis.

Initial diagnostic workup included an abdominal ultrasound (U/S), which was followed by a contrast-enhanced computed tomography (CT) scan as the previous surgery had been based on the U/S alone. The CT scan was performed to provide a more detailed assessment of the bladder wall and surrounding structures, which revealed irregular, thickened bladder walls (8 mm), but no gross mass lesion due to the previous TURBT procedure (Fig. 2). Given the history of adenocarcinoma and to assess potential metastasis, a fluorodeoxyglucose positron emission tomography (FDG-PET) was recommended by the multidisciplinary team. The FDG-PET was crucial for detecting any hypermetabolic nodular lesions suggestive of metastatic spread; however, the results revealed no abnormal focal hypermetabolic nodular lesions in the urinary bladder wall or elsewhere in the body (Fig. 3). Based on the PET scan findings and the initial TURBT biopsy, which revealed a T2-stage adenocarcinoma, a radical cystectomy was advised for definitive management.

### Therapeutic intervention

**Figure 4:**
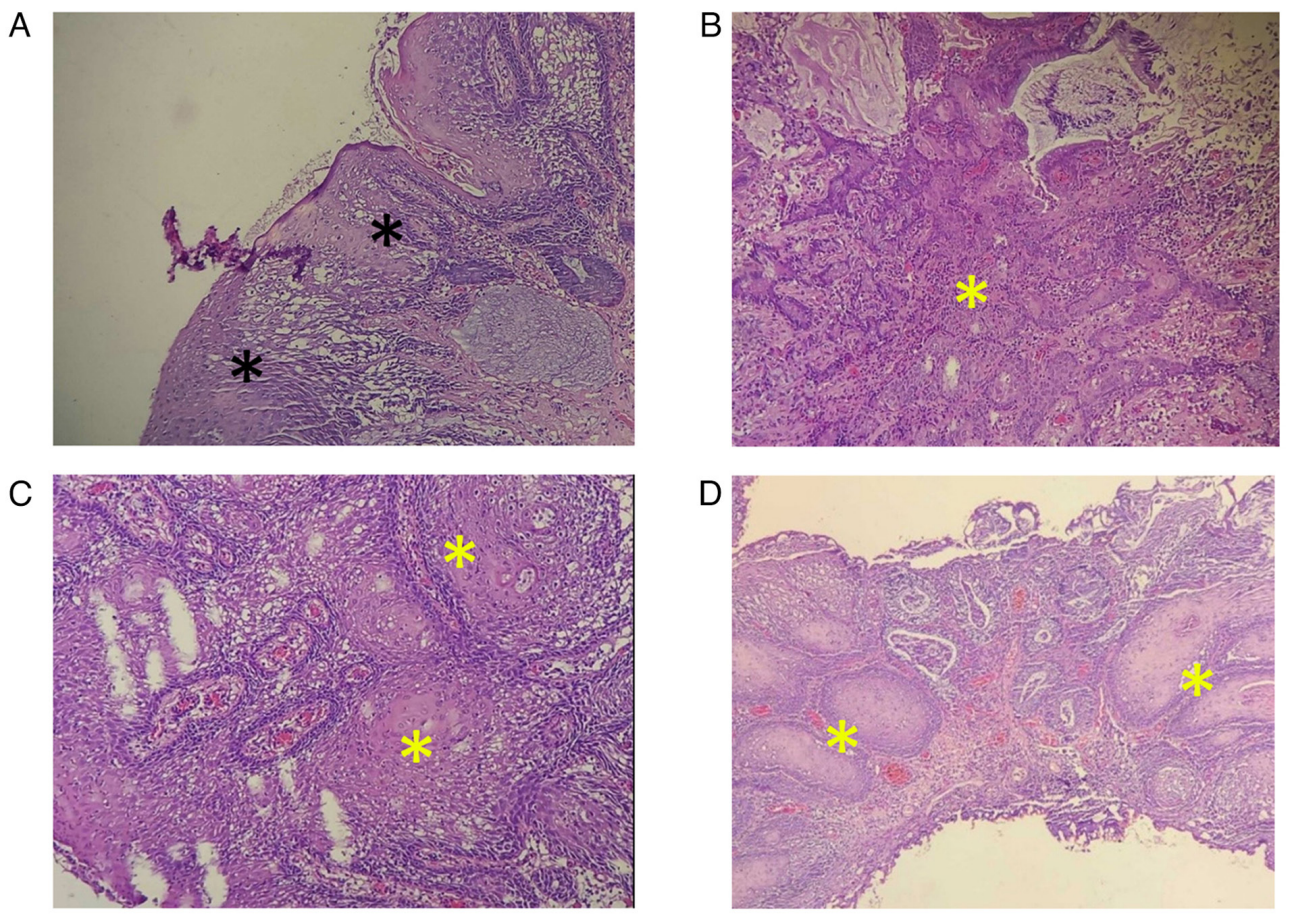
(A) Bladder mucosa with squamous metaplasia and full-thickness squamous dysplasia (black asterisks). (B-D) Stained sections indicating the irregular downward infiltration of the atypical squamous nests (yellow asterisks) into the lamina propria and deeper layers of the bladder (hematoxylin and eosin staining; magnification, x100).

The patient underwent radical cystectomy, during which gross examination revealed a shrunken bladder (7x10 cm) with a pale lesion at the trigone measuring approximately 25 mm. The excised bladder specimen was fixed in 10% neutral-buffered formalin at room temperature for 24 h before being processed using the DiaPath Donatello automated tissue processor. The tissue underwent dehydration in a graded alcohol series, clearing in xylene and paraffin infiltration, followed by embedding in paraffin wax using the Sakura Tissue-Tek embedding center. Paraffin-embedded sections, 5-µm thick, were obtained using the Sakura Accu-Cut SRM microtome, floated in a 40-50˚C water bath, and mounted on glass slides. The slides were then baked at 60-70˚C overnight before staining with hematoxylin and eosin (H&E) (Bio Optica Co.) for 1-2 min at room temperature using the DiaPath Giotto autostainer. The stained slides were examined under a light microscope (Leica Microsystems GmbH). A histopathological examination (HPE) revealed a marked ulceration at the trigone with necrosis extending into the muscle layer. Extensive squamous metaplasia throughout the bladder, SCC *in situ*, and foci of superficially invasive SCC were observed (Fig. 4). Glandular metaplasia with dysplasia was also noted, extending into the distal bladder, which exhibited significant inflammation and degenerative changes. Additionally, two perivesical nodes were found to be free of malignancy, and 18 lymph nodes were found after sectioning the right and left pelvic nodes, and all were clear of malignancy. On the TURBT, there was no residual adenocarcinoma. The final TNM stage was poorly differentiated adenocarcinoma pT2 N0 M0 and well-differentiated SCC T1 N0 M0 as a collision tumor. This combination of adenocarcinoma and SCC within the same mass is a hallmark of collision tumors, underscoring the multifocal and independent origins of each tumor type.

### Follow-up

The patient is under an extensive follow-up regimen, which includes regular urine cytology, liver function tests, and monitoring of creatinine and electrolytes. Imaging studies of the chest, urinary tract, abdomen and pelvis are also performed periodically. Additionally, annual vitamin B12 monitoring is conducted. No recurrence was observed after 1 year of follow-up.

## Discussion

While the case presented herein is unique in its specifics, the literature review highlighted several cases of urinary bladder collision tumors in patients aged 50 to 74 years, with most cases involving male patients. Common symptoms included hematuria, sometimes accompanied by urinary urgency, dysuria, or the presence of blood clots. Diagnostic imaging techniques, such as CT scans, ultrasounds and MRIs, have been frequently employed to identify the tumors and assess their extent. Surgical interventions were the primary treatment methods, including TURBT and radical cystectomy, often combined with pelvic lymph node dissection. In some instances, additional treatments such as chemotherapy or immunotherapy were utilized to manage the disease. Patient outcomes varied; while some patients experienced recurrence or metastasis, leading to further complications or mortality, others had successful surgeries and remained free from recurrence for several months to years following treatment. The majority of cases (60%) were found to result in either recurrence or mortality, while 2 cases exhibited no recurrence following an average follow-up period of 6.5 months (2,10,13-15) (Table I). By contrast, the patient in the present study remained free from recurrence during follow-up. It is noteworthy that, although the cases reviewed predominantly involve male patients, the present case pertains to a female patient, thereby contributing to the diversity in the clinical presentation of bladder collision tumors.

Collision tumors may exhibit diverse origins and growth patterns, often involving two distinct tumor types that originate independently within the same mass. This combination of adenocarcinoma and SCC in the present study within the same mass is a hallmark of collision tumors, underscoring the multifocal and independent origins of each tumor type (2). In contrast to collision tumors, composite tumors involve different cell types from the same tissues. The most frequently observed combination in bladder collision tumors, as in the present case, is the simultaneous presence of urothelial carcinoma and another type of tumor, such as adenocarcinoma, or SCC (3). Adenocarcinoma of the bladder emerges from the glandular cells of the bladder. Unlike the more prevalent transitional cell carcinoma type, it is less common. It can be in different areas of the bladder, such as the mucosal layer and deeper muscle layers (16). Cigarette smoking increases the risk of bladder cancer, as harmful chemicals in tobacco smoke are absorbed into the bloodstream and excreted through the kidneys, exposing the bladder lining to carcinogens. Bladder cancer is more common in older adults, especially men. Occupational exposures to chemicals such as aromatic amines and polycyclic aromatic hydrocarbons, found in industries such as rubber, dye, and chemical production also increase the risk. Prolonged exposure to chemicals in textiles, paints, and plastics, along with chronic urinary infections, can further increase this risk. Additionally, radiation therapy, certain diabetes medications, and chronic *Schistosoma haematobium* infection are linked to higher bladder cancer risk (17,18). The starting point of the disease is a genetic mutation, which can lead to uncontrolled cell growth and division. These mutations can be inherited or occur spontaneously during a person's lifetime; chronic inflammation and long-term inflammation in an organ can create an environment that promotes the growth of cancer cells (19). The signs and symptoms of bladder collision tumors can vary. The urine may appear pink, red, or dark brown, and the presence of blood can be intermittent or consistent; also, there may be painful urination, urinary urgency, and pelvic pain (4). In the present study, the patient was diagnosed with bladder carcinoma and was referred to the Rzgary Oncology Clinic.

The diagnosis of bladder carcinoma often involves various tests, such as urine analysis, imaging studies, and cystoscopy. A biopsy is the gold standard for the diagnosis (12). Since this type of cancer is mostly diagnosed in the advanced stages and is associated with high recurrence rates, the understanding of gene expression regulation is significant for early diagnosis and treatment. Epigenetic modifications, which alter gene expression without changing the DNA sequence, have been extensively studied as flexible and important therapeutic targets for cancer treatments. Mutations in genes such as FGFR3, PIK3CA, KDM6A and TP53 are common in bladder cancers and disrupt normal gene regulation and cell growth, leading to uncontrolled cell growth and tumor formation (20). The case described herein underwent a CT scan with IV contrast followed by a PET scan. The HPE of the specimen confirmed the diagnosis.

The management of bladder collision tumors includes cancer staging and grading, the assessment of the overall health of the patient and the consideration of the preferences of the patient. A general outline of the management options for collision bladder is TURBT, which is the standard initial treatment for non-invasive or early-stage bladder cancer, radical cystectomy, intravesical therapy and radiation therapy. Chemotherapy and immunotherapies are used in the advanced stages. Some bladder cancers with specific molecular features may be treated with targeted therapies that aim to inhibit specific abnormal proteins or pathways involved in cancer growth (21,22). This case undergone radical cystectomy as far as it was a localized early-stage disease.

Early detection and appropriate treatment are crucial for better outcomes in bladder carcinoma. Regular check-ups and prompt medical attention for concerning symptoms can improve prognosis and increase the likelihood of successful treatment. As with any type of cancer, a multidisciplinary approach involving urologists, oncologists and other healthcare specialists is often necessary to provide comprehensive care to individuals affected by bladder carcinoma (5). Although it is a rare disease, regular follow-up is necessary in cases of bladder cancer (13-16,23). The case in the present study was kept on an extensive and regular follow-up schedule by conducting urine cytology, liver function tests, serum creatinine and electrolytes.

In conclusion, collision tumor of the bladder is a rare disease; extensive radiological study and careful examination of the biopsy are necessary to diagnose the condition.

## Figures and Tables

**Table I:** Summary of several reported cases of urinary bladder collision tumors in the literature.

					Imaging used for diagnosis			Tumor characteristics					
Author(s)	Country, year of publication	Age, years	Sex	Presentation	U/S	CT/MRI	Management	Location	Gross-section size	HPE	Adjuvant chemotherapy	Recurrence	(Refs.)
Gandhi *et al*	India, 2017	73	Male	Hematuria for15 days	Revealed a bladder mass	Showed a bladder mass measuring 5.6x2.4 cm with significant bilateral pelvic lymphadenopathy	Radical cystoprostatectomy with bilateral pelvic lymph node dissection and resection of a sigmoid colon serosal deposit	Posterior wall and dome of the bladder	6x4.5x3 cm	Large cell neuroendocrine carcinoma with adenocarcinoma component	Recommended but refused by the patient	Patient died 4 months post-surgery	(2)
Jiang *et al*	China, 2023	64	Male	Urinary urgency, frequent urination, dysuria, and recurrent hematuria.	Not applicable	Irregular and abnormal signal shadow in the bladder, measuring 8.4x6 cm, and abnormal signal in the central gland of the prostate, Chemo-therapy (gemcitabine + cisplatin) and immunotherapy (tislelizumab)	Multiple TURBT procedure+ Radical cystectomy with pelvic lymph node resection and cutaneous ureterostomy in May 2021	Bladder and prostate involvement	Up to 8.4x6 cm	Collision tumor with small cell small cell carcinoma (SCC) and high-grade urothelial carcinoma	Recommended but the patient had difficulty adhering to the treatment schedule due to economic reasons	Neck and mediastinum lymph nodes metastases; patient died of COVID-19	(10)
Brinton *et al*	United States, 1970	50	Male	Gross, total, painless hematuria for 4 days	Not applicable	Not applicable	TURBT followed by total cystectomy and ileal loop urinary diversion.	Superior to the right ureteral orifice	2 cm (polypoid tumor)	Carcinosarcoma of the bladder with muscle invasion	Not applicable	No recurrence; patient alive and well 5 months postsurgery.	(13)
Chen *et al*	China, 2022	74	Male	Gross hematuria for 3 months worsening in the past week, occasionally accompanied by blood clots.	Revealed a substan tial mass in the bladder measuring 3.1x 2.4x2.1 cm with a rich blood flow signal. Additionally, the prostate was enlarged with intense light spots	CT scan: Indicated focal thickening of the left posterior wall of the bladder with a cauliflower-shaped soft-tissue density shadow protruding into the bladder. On contrast-enhanced CT, the lesion was mildly to moderately enhanced, suggestive of a space-occupying lesion consistent with bladder cancer	TURBT followed by total cystectomy	Left posterior wall of the bladder, 1 cm lateral to the left ureteral opening	A volume of mass was 3.0x2.0x 2.0 cm	Collision tumor between primary malignant melanoma and high-grade non-invasive urothelial papillary carcinoma.	No chemotherapy, immunotherapy, or gene therapy was administered.	Patient died of systemic metastasis 31 months after surgery	(14)
Kitazawa *et al*	Japan, 1985	67	Male	Occasional small amounts of gross hematuria and dysuria, with discharge of a fingertip-sized mass.	Not applicable	Not applicable	TURBT, section by section.	Right posterior wall of the bladder	2.1x1.8 cm; the specimen weighed 4.0 grams.	Giant cell tumor associated with transitional cell carcinoma.	Not applicable	No recurrence or metastasis 8 months after TURBT.	(15)

US, ultrasound; CT, computed tomography, MRI, magnetic resonance imaging; HPE, histopathological examination; TURBT, transurethral resection of bladder tumor.

## Data Availability

The data generated in the present study may be requested from the corresponding author.
